# The Blockade of TACE-Dependent EGF Receptor Activation by Losartan-Erlotinib Combination Attenuates Renal Fibrosis Formation in 5/6-Nephrectomized Rats Under Vitamin D Deficiency

**DOI:** 10.3389/fmed.2020.609158

**Published:** 2021-01-05

**Authors:** Janaína Garcia Gonçalves, Daniele Canale, Ana Carolina de Bragança, Antonio Carlos Seguro, Maria Heloisa Massola Shimizu, Rildo Aparecido Volpini

**Affiliations:** ^1^Laboratorio de Investigacao Medica 12, Faculdade de Medicina, Universidade de São Paulo, São Paulo, Brazil; ^2^Laboratorio de Investigacao Medica 12, Hospital das Clinicas HCFMUSP, Faculdade de Medicina, Universidade de São Paulo, São Paulo, Brazil

**Keywords:** chronic kidney disease, TACE, renal fibrosis, vitamin D deficiency, erlotinib, experimental model, EGF receptor (EGFR), EGF (epidermal growth factor)

## Abstract

Chronic kidney disease (CKD) has been considered a major public health issue. In addition to cardiovascular diseases and infections, hypovitaminosis D has been considered a non-traditional aggravating factor for CKD progression. Interstitial fibrosis is a hallmark of CKD strongly correlated with deterioration of renal function. Transforming growth factor β (TGF-β) is the major regulatory profibrotic cytokine in CKD. Many injurious stimuli converge on the TGF-β pathway, which has context-dependent pleiotropic effects and interacts with several related renal fibrosis formation (RFF) pathways. Epidermal growth factor receptor (EGFR) is critically involved in CKD progression, exerting a pathogenic role in RFF associated with TGF-β-related fibrogenesis. Among others, EGFR pathway can be activated by a disintegrin and a metalloproteinase known as tumor necrosis factor α-converting enzyme (TACE). Currently no effective therapy is available to completely arrest RFF and slow the progression of CKD. Therefore, we investigated the effects of a double treatment with losartan potassium (L), an AT1R antagonist, and the tyrosine kinase inhibitor erlotinib (E) on the alternative pathway of RFF related to TACE-dependent EGFR activation in 5/6-nephrectomized rats under vitamin D deficiency (D). During the 90-day protocol, male Wistar rats under D, were submitted to 5/6 nephrectomy (N) on day 30 and randomized into four groups: N+D, no treatment; N+D+L, received losartan (50 mg/kg/day); N+D+E, received erlotinib (6 mg/kg/day); N+D+L+E received losartan+erlotinib treatment. N+D+L+E data demonstrated that the double treatment with losartan+erlotinib not only blocked the TACE-dependent EGF receptor activation but also prevented the expression of TGF-β, protecting against RFF. This renoprotection by losartan+erlotinib was corroborated by a lower expression of ECM proteins and markers of phenotypic alteration as well as a lesser inflammatory cell infiltrate. Although erlotinib alone has been emerging as a renoprotective drug, its association with losartan should be considered as a potential therapeutic strategy on the modulation of RFF.

## Introduction

Chronic kidney disease (CKD) has been considered a major public health issue worldwide associated with serious consequences. Among others, CKD encompasses increased risk of mortality, end-stage renal disease (ESRD), accelerated cardiovascular disease (CVD), mineral and bone disorders, adverse metabolic and nutritional consequences, infections, reduced cognitive function, and increased risk of acute kidney injury (AKI) (1, 2). Estimates from different parts of the world reveal an increasing incidence and prevalence of CKD, which is usually associated with risk factors such as the global increasing prevalence of diabetes, hypertension, obesity and CVD (2, 3). Those major risk factors account for only half of the causes of mortality and several studies have been highlighting the vitamin D [25(OH)D] status as a non-traditional aggravating factor for renal diseases (4, 5). In previous studies involving some experimental models, we showed that vitamin D deficiency (VDD) exacerbated the renal morphological alterations, including the interstitial enlargement as a result of renal fibrosis and inflammatory infiltrate (4–9). In addition, those alterations were associated with activation of renin-angiotensin-aldosterone system (RAAS) (4–7, 9) and increased expression of transforming growth factor β (TGF-β) under downregulation of vitamin D receptor (VDR) in the renal tissue (4, 5).

Interstitial fibrosis is a hallmark of CKD and strongly correlates with deterioration of renal function (10). The classical experimental 5/6 nephrectomy is a well-established model of renal tubulointerstitial fibrosis. Histological studies concerning this model show a complex response firstly identified by hypertrophic and quiescent phases, with minimal histological alterations damage of the remnant kidney. The last phase, or end stage, is characterized by a progressive and irreversible decline in renal function, usually associated with histomorphological changes (4, 11). The fibrogenic response relies basically on fibroblasts, which are considered to be the effector cells in fibrogenesis (12). Among others, angiotensin II (AII) and TGF-β are major fibrogenic factors that mediate fibrogenesis in the kidney (10, 13). Basically, AII promotes renal fibrosis directly via the angiotensin type 1 receptor (AT1R) or activation of TGF-β pathway (10, 14). TGF-β is a potent profibrotic factor that directly stimulates the expression of many extracellular matrix (ECM) proteins in renal cells and promotes epithelial-mesenchymal transition (EMT) in the kidney (10, 15). The TGF-β canonical signaling *via Smads* has a pivotal function in the development of renal fibrosis although signals through non-canonical pathways also exert an important role in the progression of kidney disease (16, 17). Those non-canonical or alternative TGF-β pathways usually act in a *Smad*-independent manner and comprise the signaling with, among others, connective tissue growth factor (CTGF), platelet-derived growth factor (PDGF), epidermal growth factor (EGF) and *Wnt* pathways (17, 18).

Over the past decades, increasing evidence indicates that epidermal growth factor receptor (EGFR) is critically involved in the progression of CKD, exerting a pathogenic role in renal fibrosis formation (RFF) (19, 20). EGFR is endogenously expressed in several cell types and exerts an important control on cell cycle, cell migration, metabolism and survival, and cellular proliferation and differentiation (21). Both EGFR and its ligands, including EGF and transforming growth factor α (TGF-α), are plentifully expressed along the nephron (20, 21). Upon activation by its ligands, the autophosphorylation of several tyrosine residues in EGFR occurs, which elicits downstream activation and signaling (19–21). Previous experimental studies have provided convincing evidence that dysregulated EGFR signaling is involved in mediating renal fibrogenesis in CKD (19–23). Moreover, a number of reports have been suggesting that EGFR is associated with TGF-β-related fibrogenesis (20, 24, 25).

In addition to the well-described role of AII acting on pathways associated to RFF, other effects of this peptide may be mediated by the activation of a disintegrin and a metalloproteinase (ADAM)17 [also termed tumor necrosis factor α (TNF-α)-converting enzyme: TACE] (21, 22). Upon AII signaling, TACE becomes active and consequently activates TGF-α, thereby initiating EGFR signaling (19, 21). Furthermore, EGFR-stimulated RFF can also be AII-independent, as EGF stimulation alone could induce markers of fibrosis in SV 40 cells (19, 26). In 2018, Yamamoto et al. (20) demonstrated that EGFR blocking by erlotinib, a tyrosine kinase inhibitor (TKI) of EGFR, not only protected against renal fibrosis in 5/6-nephrectomized rats but also decreased the inflammation, highlighting the renoprotective effects of this TKI. Moreover, some studies show that TKIs have been applied in interventional studies of renal fibrosis in mice established by chronic AII infusion and unilateral ureteral obstruction (24, 25). However, the effects of a simple pharmacological EGFR inhibition have not been sufficient to stop RFF and yet, not clearly elucidated. Here, we investigated the effects of a dual treatment involving losartan, an AT1R antagonist, and erlotinib on the alternative pathway of RFF related to TACE-dependent EGFR activation. Moreover, we attempted to study the efficiency of the dual treatment losartan+erlotinib in a model of 5/6 nephrectomy aggravated by VDD.

## Materials and Methods

### Animals and Experimental Protocol

Thirty-five adult male Wistar rats (*Rattus novergicus*), weighing 180–200 g, were used in this study. All rats were obtained from a local facility at the Institute of Biomedical Sciences-University of São Paulo. The experimental procedures were specifically approved by the local Research Ethics Committee (CEUA, registration 122/16) in accordance with our institutional guidelines and well-established international standards for the care and use of laboratory animals. All surgeries were performed under appropriate anesthesia, and all efforts were made to minimize animal suffering. During the 90-day protocol, all animals were maintained under standard laboratory conditions, with free access to vitamin D-free diet (0.4% calcium and 0.4% phosphate) (MP Biomedicals, Irvine, CA) and tap water.

On day 30, 5/6 nephrectomy (N) was performed in a single-step procedure in all vitamin D-deficient (D) rats. A suprapubic incision was done under 2,2,2-Tribromoethanol anesthesia [250 mg/Kg body weight (BW)]. After that, the right kidney was removed, and two or three branches of the left renal artery were ligated, resulting in the infarction of two-thirds of the left kidney and the incision was sutured immediately.

On day 37, all rats were randomly assigned to four groups: N+D (*n* =10)—untreated rats; N+D+L (*n* = 8)—rats under losartan (L) treatment (losartan potassium 50 mg/kg/day) diluted in drinking water; N+D+E (*n* = 7)—rats treated with a daily intraperitoneal erlotinib (E) injection (6 mg/kg/day) (Erlotinib, Cayman Chemical, Ann Arbor, MI) dissolved in the co-solvent dimethyl sulfoxide (Merck, Darmstadt, Germany); N+D+L+E (*n* = 10)—rats concomitantly treated with losartan+erlotinib (L+E) as described above.

### Inulin Clearance and Hemodynamic Studies

Sixty days after 5/6 nephrectomy, all animals were anesthetized with sodium thiopental (50 mg/Kg BW) and placed on a temperature-regulated surgical table. The trachea was cannulated (PE-240 catheter) and spontaneous breathing was maintained. The left jugular vein was cannulated (PE-60 catheter) for infusion of inulin and other fluids. To monitor mean arterial pressure (MAP) and collect blood samples, the right carotid artery was catheterized with a PE-50 catheter. We assessed MAP with a data acquisition system (MP100; Biopac Systems, Santa Barbara, CA). To collect urine samples, the urinary bladder was cannulated (PE-240 catheter) after a suprapubic incision. Following completion of the cannulation surgical procedure, a loading dose of inulin (100 mg/Kg BW diluted in 1 mL of 0.9% saline) was administered through the jugular vein. Subsequently, a constant infusion of inulin (10 mg/kg BW in 0.9% saline) was started and continued at a 0.04 mL/min flow throughout the whole experiment. Three urine samples were collected at 30-min intervals. Blood samples were obtained at the beginning and at the end of the experiment. Inulin clearance values represent the mean of three periods. Blood and urine inulin were determined by the anthrone method.

### Biochemical Parameters

We collected blood samples after the clearance studies to assess plasma levels of 25-hydroxyvitamin D [25(OH)D], parathormone (PTH), fibroblast growth factor 23 (FGF-23), aldosterone, phosphate (P_P_) and calcium (P_Ca_). We assessed 25(OH)D, PTH, FGF-23 and aldosterone by enzyme-linked immunosorbent (ELISA) using the following commercial kits: 25-Hydroxyvitamin D (ALPCO, Salem, NH); Rat Intact PTH and Mouse/Rat Intact FGF-23 (Immutopics, Inc., San Clemente, CA); and Aldosterone (Enzo Life Sciences, Farmingdale, NY). P_P_ and P_Ca_ were evaluated by colorimetric assay (Labtest, Lagoa Santa/MG, Brazil).

### Tissue Sample Preparation

Following clearance experiment, we perfused the remnant kidney of each animal with phosphate-buffered solution (PBS, pH 7.4). Fragments of the renal tissue were frozen in liquid nitrogen and stored at −80°C for Western blotting and ELISA experiments. Another sample of the same renal tissue was fixed in methacarn solution (60% methanol, 30% chloroform, 10% glacial acetic acid) for 24 h and in 70% alcohol thereafter. Kidney blocks were embedded in paraffin and cut into 4-μm sections for histology and immunohistochemistry studies.

### Total Protein Isolation

Renal cortex samples were homogenized in ice-cold isolation solution (200 mM mannitol, 80 mM HEPES, and 41 mM KOH, pH 7.5) containing a protease inhibitor cocktail (Sigma Chemical Company, St. Louis, MO) using a tissue homogenizer (Tissue Master TM125, Omni International, Kennesaw, GA). Homogenates were centrifuged at 4000 × rpm for 30 min at 4°C to remove nuclei and cell debris. Supernatants were isolated, and protein was quantified by Bradford assay (Bio-Rad Laboratories, Hercules, CA). We used these samples to run Western blot and ELISA experiments.

### Western Blot Assays

For Western blot analysis, 100 μg of renal cortex protein were separated on SDS-polyacrylamide minigels by electrophoresis (27). After transfer by electroelution to PVDF membranes (GE Healthcare Limited, Little Chalfont, UK), blots were blocked for 1 h with 5% non-fat dry milk in tris-buffered saline solution. Blots were then incubated overnight with primary antibodies for: TGF-β1 (1/250; Santa Cruz Biotechnology, Dallas, TX) and p-EGFR (1/100, Cell Signaling Technologies, Danvers, MA). The labeling was visualized with a horseradish peroxidase-conjugated secondary antibody (1/2,000 anti-rabbit IgG, Sigma Chemical, St. Louis, MO) and enhanced chemiluminescence (ECL) detection (GE Healthcare Limited, Little Chalfont, UK). Kidney protein levels were further analyzed with a gel documentation system (Alliance 4.2; Uvitec, Cambridge, UK) and the software Image J for *Windows* (Image J—NIH Image). We used densitometry to quantitatively analyze the protein levels, normalizing the bands to β-actin expression (1/10,000 anti β-actin, Sigma Chemical, St. Louis, MO).

### ELISA in Renal Tissue

We assessed Collagen Type III (Col III) (#MBS2023591), TGF-β1 (#MBS824788), and EGF (#MBS824918) in renal cortex samples by ELISA using commercial kits (MyBiosource, San Diego, CA), following the instructions provided by the manufacturer. The absorbances were obtained using the Epoch/2 microplate reader (Biotek Instruments, Winooski, VE).

### Light Microscopy

Four-micrometer histological sections of kidney tissue were stained with Masson's trichrome and examined under light microscopy. We evaluated the fractional interstitial area (FIA) by analyzing tubulointerstitial enlargement. For histomorphometry, the images obtained by a microscopy (Axioskop 40, Carl Zeiss, Munich, Germany) were captured on a computer screen *via* an image analyzer software (ZEN, Carl Zeiss, Munich, Germany). For FIA evaluation, we analyzed 30 grid fields (0.09 mm^2^ each) per kidney cortex. The interstitial areas were manually demarcated and the proportion of each analyzed field was determined after excluding the glomeruli. In order to minimize bias during the morphometric analysis, the observer was blinded to the treatment groups.

### Immunohistochemical Analysis

Immunohistochemistry were performed on 4-μm-thick paraffinized kidney sections mounted on 2% silane-coated glass slides. We used the following antibodies: (1/100) mouse monoclonal to CD68 (ED1) (AbD Serotec, Oxford, UK); (1/2,000) rabbit polyclonal to mannose receptor (CD206) and rabbit monoclonal to fibronectin (Abcam, Cambridge, MA); (1/50) mouse monoclonal to CD3 (T cells) and (1/50) mouse monoclonal to vimentin (DAKO, Glostrup, Denmark); (1/500) rabbit polyclonal to TGF-β1, (1/30) mouse monoclonal to TGF-α and (1/25) rabbit polyclonal to TACE (Santa Cruz Biotechnologies, Dallas, TX); (1/100) rabbit polyclonal to EGFR (Cell Signaling Technology, Danvers, MA); (1/200) mouse monoclonal to α-smooth muscle actin (α-SMA) (Millipore, Billerica, MA). We subjected the kidney tissue sections to immunohistochemical (IHC) reaction according to the protocol for each primary antibody. Reaction products were detected by avidin-biotin-peroxidase (Vector Laboratories, Burlingame, CA) and the staining was developed with 3,3-diaminobenzidine (Sigma, St. Louis, MO) in the presence of hydrogen peroxide. Counterstaining was with Harris' hematoxylin. We analyzed 40–50 renal cortex fields (0.09 mm^2^) to evaluate the immunoreactions. By computerized histomorphometry, volume ratios of positive areas of renal tissue (%), determined by the color limit, were obtained by an image analyzer software (ZEN, Carl Zeiss, Munich, Germany) on a computer coupled to a microscope (Axioskop 40; Carl Zeiss) and to a digital camera (4).

### Statistical Analysis

One-way ANOVA with paired comparisons according to the Newman-Keuls test was applied in this study by using GraphPad Prism 5.0 software (GraphPad Software, La Jolla, CA). All quantitative data were expressed as mean ± SEM (standard error of the mean). *P*-values < 0.05 were considered statistically significant.

## Results

### Glomerular Filtration Rate and Mean Arterial Blood Pressure

Renal function and hemodynamic parameters obtained 60 days after 5/6 nephrectomy are given in Table 1. We observed a partial recovery of GFR (mL/min/100g) in N+D+L and N+D+L+E groups in comparison to N+D rats. N+D+E group presented a slight tendency to recover renal function, however, without significance. Regarding hemodynamic parameters, we found significant lower levels of MAP (mmHg) in the N+D+L, N+D+E and N+D+L+E groups than in the N+D group. In addition, MAP levels in the N+D+E group were statistically higher than in the N+D+L and N+D+L+E groups. Thus, losartan treatment alone or associated with erlotinib attenuated GFR and MAP alterations observed after 5/6 nephrectomy.

**Table 1:** Renal function, hemodynamic and biochemical parameters evaluated 60 days after 5/6 nephrectomy (N) in vitamin D deficient rats (D) treated with losartan (L), erlotinib (E) or losartan+erlotinib (L+E).

	**N+D**	**N+D+L**	**N+D+E**	**N+D+L+E**
GFR (mL/min/100g BW)	0.39 ± 0.02	0.63 ± 0.04^a^	0.50 ± 0.03	0.60 ± 0.04^b^
MAP (mmHg)	163 ± 4	108 ± 2^a^	147 ± 4^c^^d^	107 ± 3^a^^g^
25(OH)D (ng/mL)	<0.44	<0.44	<0.44	<0.44
PTH (pg/mL)	1,296 ± 348	760 ± 118	602 ± 168	365 ± 56^b^
FGF-23 (pg/mL)	529.8 ± 40.2	458.6 ± 43.6	458.7 ± 34.1	403.7 ± 37.5
Proteinuria (mg/24 h)	24.5 ± 2.7	13.8 ± 1.3^a^	15.8 ± 1.5^a^	11.5 ± 0.7^a^
Aldosterone (pg/mL)	3,692 ± 390	1,496 ± 319^a^	3,266 ± 644^f^	1,650 ± 252^b^^i^
P_Catotal_ (mg/dL)	7.50 ± 0.14	7.49 ± 0.11	7.77 ± 0.11	7.78 ± 0.18
P_P_ (mg/dL)	5.4 ±0.9	5.0 ± 0.2	4.2 ± 0.3	4.8 ± 0.1

GFR, inulin clearance; BW, body weight; MAP, mean arterial pressure; 25(OH)D, 25-hydroxyvitamin D; PTH, parathormone; FGF-23, fibroblast growth factor 23; P_Catotal_, plasma calcium concentration; P_P_, plasma phosphorus concentration. Values are mean ± SEM.

a p < 0.001,

b p < 0.01,

c p < 0.05 vs. N+D;

d p < 0.001,

f p < 0.05 vs. N+D+L;

g p < 0.001,

i *p < 0.05 vs. N+D+E. N, 5/6-nephrectomized rats; D, vitamin D-deficient rats; L, losartan-treated rats; E, erlotinib-treated rats; L+E, rats concomitantly treated with losartan+erlotinib. N+D (n = 10); N+D+L (n = 8); N+D+E (n = 7); N+D+L+E (n = 10)*.

### Biochemical Parameters

All data regarding biochemical parameters are shown in Table 1. As previously described, our animals fed a vitamin D-free diet for 90 days. Predictably, all groups of animals presented undetectable plasma levels (<0.44 ng/mL) of 25(OH)D at the end of the experimental period. Concerning intact PTH (pg/mL), N+D+L+E rats presented significant lower plasma levels of this hormone than N+D rats. In addition, we noted a downward in PTH levels in N+D+L and N+D+E groups, however, without significance. Despite the changes observed in PTH, we did not find any significant difference in plasma levels of FGF-23 among the animal groups. Our data on proteinuria (mg/24 h) showed that treatments with losartan, erlotinib or both losartan+erlotinib prevented the increase of proteinuria in these groups in comparison to N+D group. Corroborating our MAP data, we found significant lower plasma levels of aldosterone (pg/mL) in the rats under losartan treatment (alone or associated) than in the N+D and N+D+E rats. Lastly, we also performed the measurement of plasma levels of calcium and phosphorus (mg/dL), which did not present statistical difference among the groups.

### The Dual Treatment Losartan+Erlotinib Strongly Attenuates Renal Fibrosis Formation

**Figure 1:**
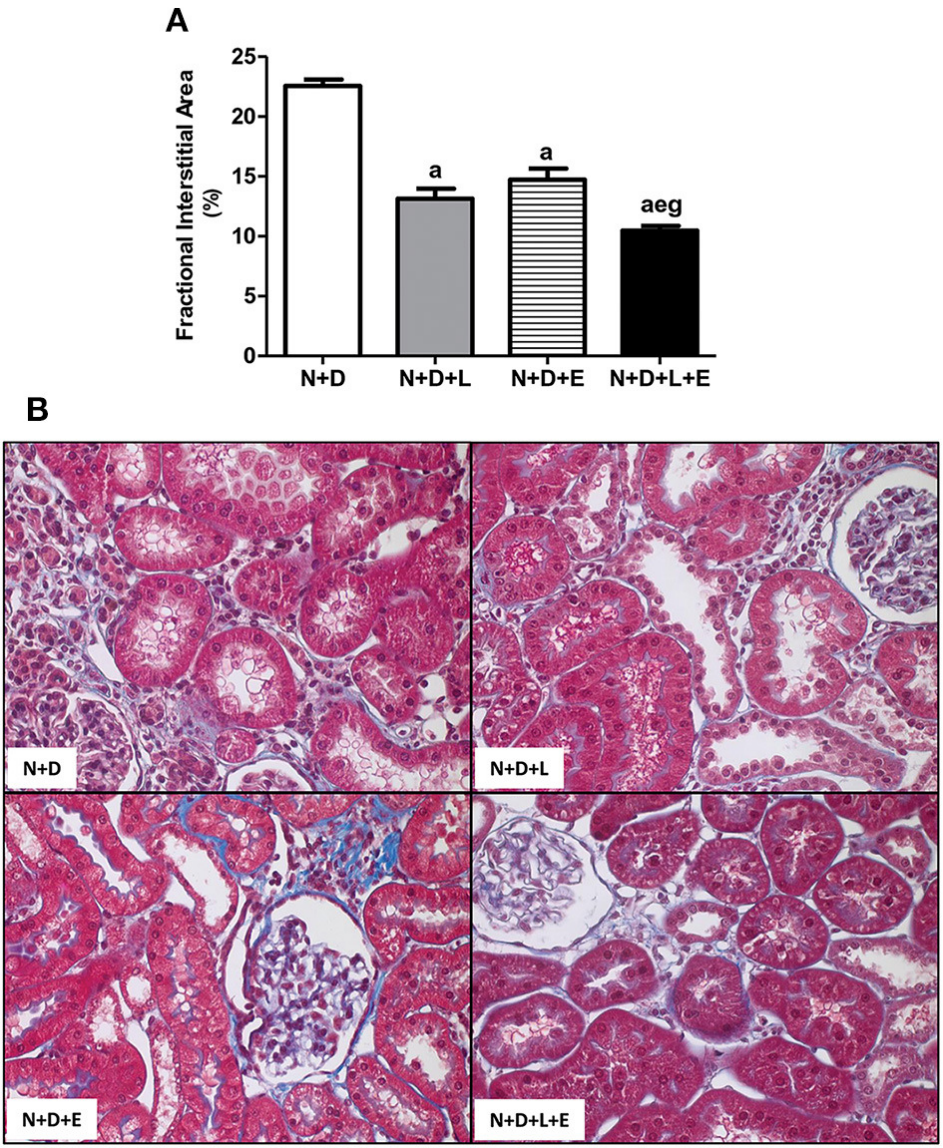
Fractional interstitial area (FIA) in the renal cortex. **(A)** Bar graph of FIA volumes evaluated 60 days after 5/6 nephrectomy in vitamin D-deficient rats treated with losartan, erlotinib or losartan+erlotinib. **(B)** Representative photomicrographs of the renal cortex from a N+D, N+D+L, N+D+E, and N+D+L+E rat (x400). Note that the FIA is significantly smaller in the N+D+L+E than in the other groups. Data are mean ± SEM. ^a^*p* < 0.001 vs. N+D; ^e^*p* < 0.01 vs. N+D+L; ^g^*p* < 0.001 vs. N+D+E. N, 5/6-nephrectomized rats; D, vitamin D-deficient rats; L, losartan-treated rats; E, erlotinib-treated rats; L+E, rats concomitantly treated with losartan+erlotinib. N+D (*n* = 10); N+D+L (*n* = 8); N+D+E (*n* = 7); N+D+L+E (*n* = 10).

Five-sixths nephrectomy is a well-known experimental model of CKD whose main features include the presence of focal segmental glomerulosclerosis and tubulointerstitial fibrosis (4, 28). Under light microscopy, we observed classical alterations related to 5/6 nephrectomy model such as interstitial fibrosis, tubular atrophy and dilatation, and inflammatory cell infiltrate in the renal cortex of all groups of animals. In addition, our morphometric studies performed to evaluate the FIA (%) of the renal cortex revealed a significant and larger diffuse interstitial expansion and renal fibrosis in the N+D group than in the other groups. The monotherapy with losartan reduced the interstitial expansion about 42% in N+D+L group while erlotinib alone was responsible for a smaller FIA (~35%) when we compared both groups to N+D group (Figure 1). Most importantly, we observed a remarkable and significant protection concerning interstitial enlargement in N+D+L+E group compared to the other groups. In other words, the dual treatment losartan+erlotinib provided a prevention of interstitial expansion and subsequently RFF in the N+D+L+E group (Figure 1). Therefore, our results show that the double therapy losartan+erlotinib is more effective than the monotherapies concerning the attenuation of RFF in our 5/6 nephrectomy model of CKD under hypovitaminosis D.

### The Association Losartan+Erlotinib Markedly Decreased the Expression of TGF-β

**Figure 2:**
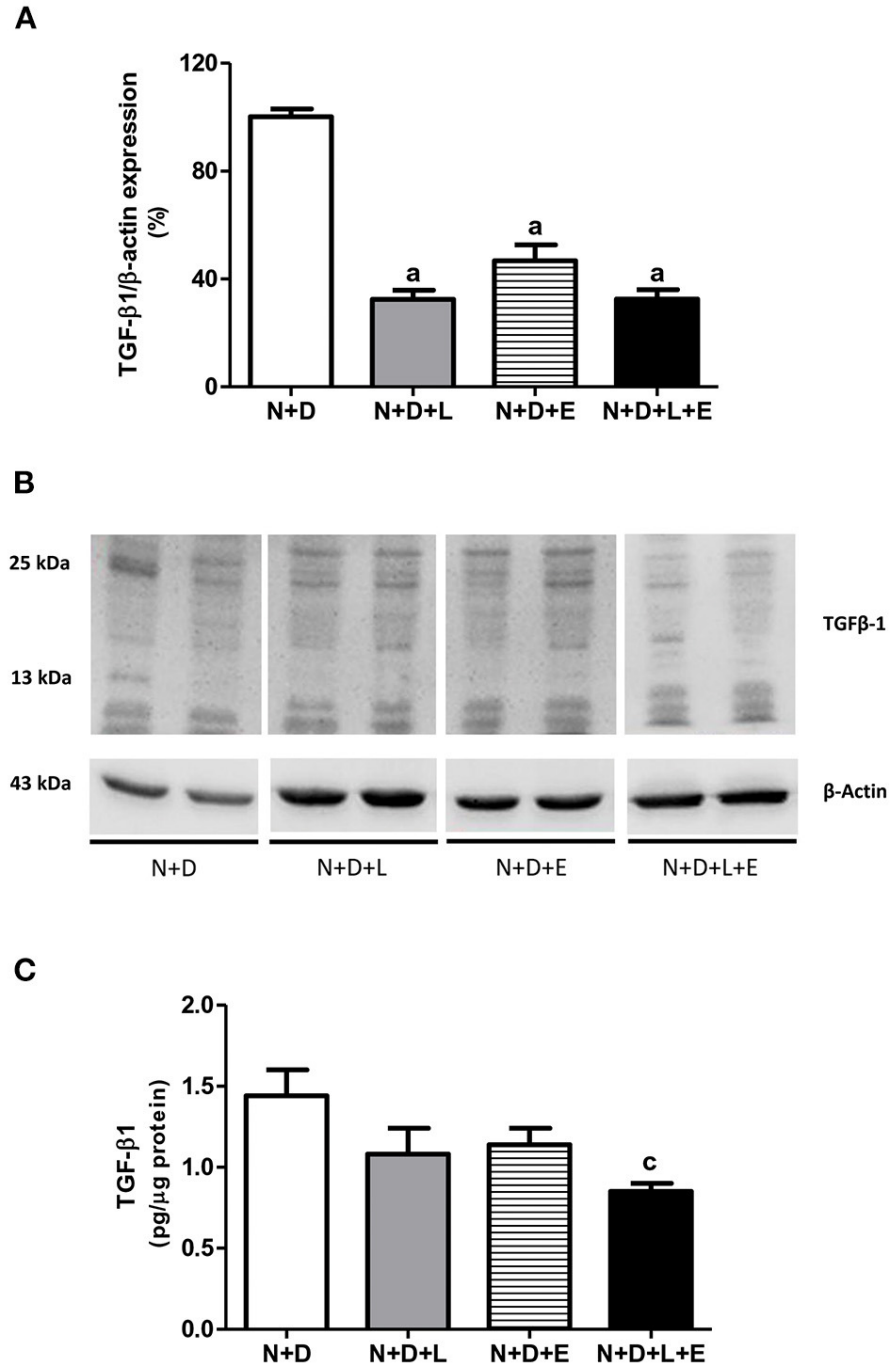
Assessment of transforming growth factor-β1 (TGF-β1) amounts in the renal cortex evaluated 60 days after 5/6 nephrectomy in vitamin D-deficient rats treated with losartan, erlotinib or losartan+erlotinib. **(A)** Semiquantitative immunoblotting for TGF-β1 with respective densitometric analysis of samples from N+D, N+D+L, N+D+E, and N+D+L+E groups. **(B)** Representative immunoblots which reacted with anti-TGF-β1 revealing both 13 and 25 kDa bands. **(C)** Quantitative amount of TGF-β1 evaluated by ELISA in the N+D, N+D+L, N+D+E, and N+D+L+E groups. Data are mean ± SEM. ^a^*p* < 0.001, ^c^*p* < 0.05 vs. N+D. N, 5/6-nephrectomized rats; D, vitamin D-deficient rats; L, losartan-treated rats; E, erlotinib-treated rats; L+E, rats concomitantly treated with losartan+erlotinib. N+D (*n* = 10); N+D+L (*n* = 8); N+D+E (*n* = 7); N+D+L+E (*n* = 10).

Although the focus of this work is on TACE-dependent EGFR activation as an alternative pathway of RFF, we have also been committed to study the participation of TGF-β on renal fibrosis after treatments with losartan, erlotinib or both losartan+erlotinib. Our Western Blotting data showed a significant lower TGF-β1 renal expression (%) in rats under losartan, erlotinib or both losartan+erlotinib treatments than in the N+D rats (Figure 2). Although we have observed a reduction (~20–25%) in TGF-β1 (pg/μg prot) in N+D+L and N+D+E groups, only N+D+L+E group presented a significant lower amount (~41%) of this cytokine in comparison to N+D group (Figure 2). Moreover, our IHC results regarding TGF-β1 (%) showed that both losartan- or erlotinib-alone treatments reduced the expression of this peptide in the renal epithelial cells from N+D+L (1.90 ± 0.29) and N+D+E (1.84 ± 0.45) groups compared to N+D group (3.92 ± 0.39). Corroborating Western Blotting and ELISA data, our IHC results showed that the dual treatment losartan+erlotinib significantly reduced the TGF-β1 renal expression in the N+D+L+E group (0.83 ± 0.15) in comparison to the other groups. Therefore, our data show that losartan or erlotinib monotherapies partially prevented the expression of TGF-β1 in the renal tissue. Most importantly, the dual treatment losartan+erlotinib induced a remarkable blockade on TGF-β1 renal expression in the N+D+L+E rats.

### The Combined Treatment Losartan+Erlotinib Efficiently Blocked the Alternative Pathway of RFF Related to TACE-Dependent EGFR Activation

**Figure 3:**
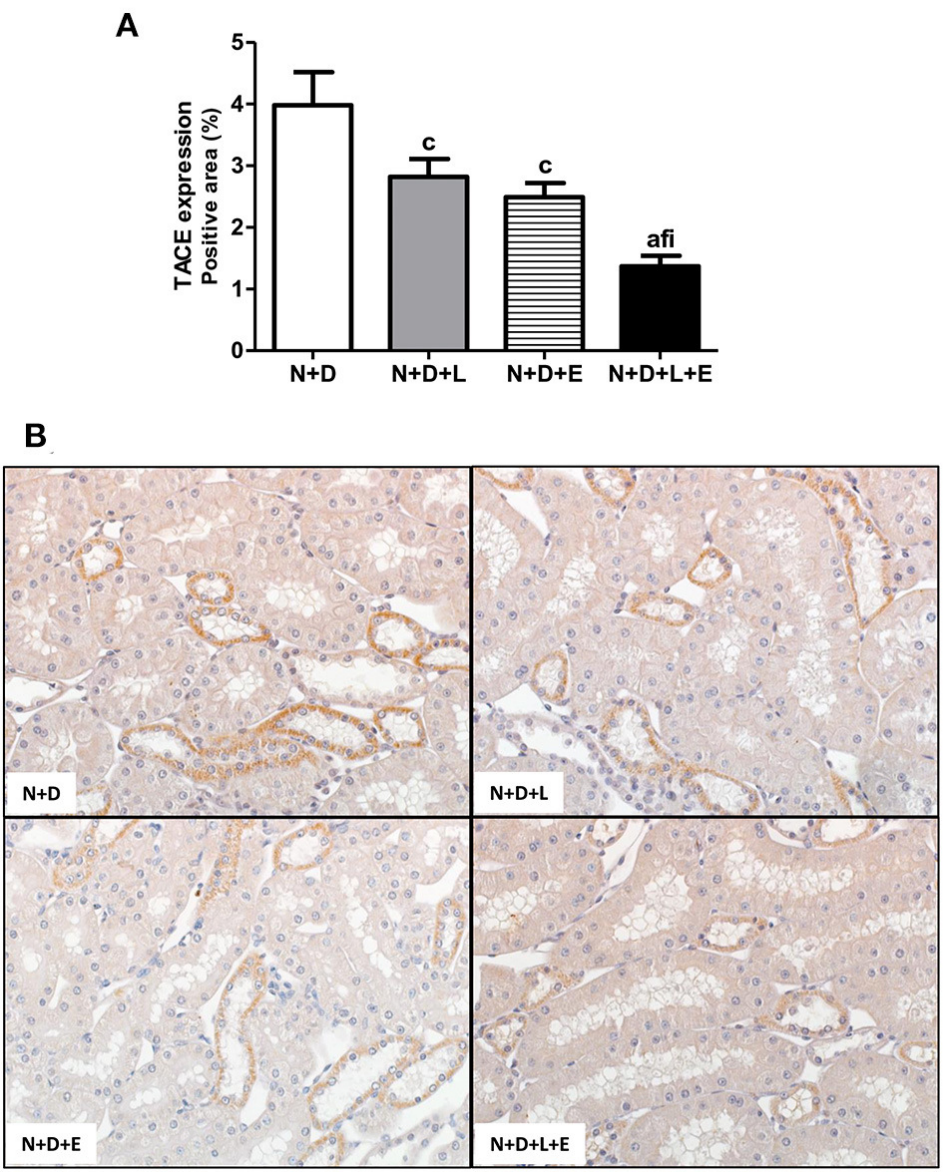
Immunohistochemical analysis of tumor necrosis factor α-converting enzyme (TACE) expression in the renal cortex. **(A)** Bar graph of TACE expression evaluated 60 days after 5/6 nephrectomy in vitamin D-deficient rats treated with losartan, erlotinib or losartan+erlotinib. **(B)** Representative photomicrographs of immunostaining for TACE in the renal cortex from a N+D, N+D+L, N+D+E, and N+D+L+E rat (x400). Note that TACE expression is significantly lower in the N+D+L+E than in the other groups. Data are mean ± SEM. ^a^*p* < 0.001, ^c^*p* < 0.05 vs. N+D; ^f^*p* < 0.05 vs. N+D+L; ^i^*p* < 0.05 vs. N+D+E. N, 5/6-nephrectomized rats; D, vitamin D-deficient rats; L, losartan-treated rats; E, erlotinib-treated rats; L+E, rats concomitantly treated with losartan+erlotinib. N+D (*n* = 10); N+D+L (*n* = 8); N+D+E (*n* = 7); N+D+L+E (*n* = 10).

**Figure 4:**
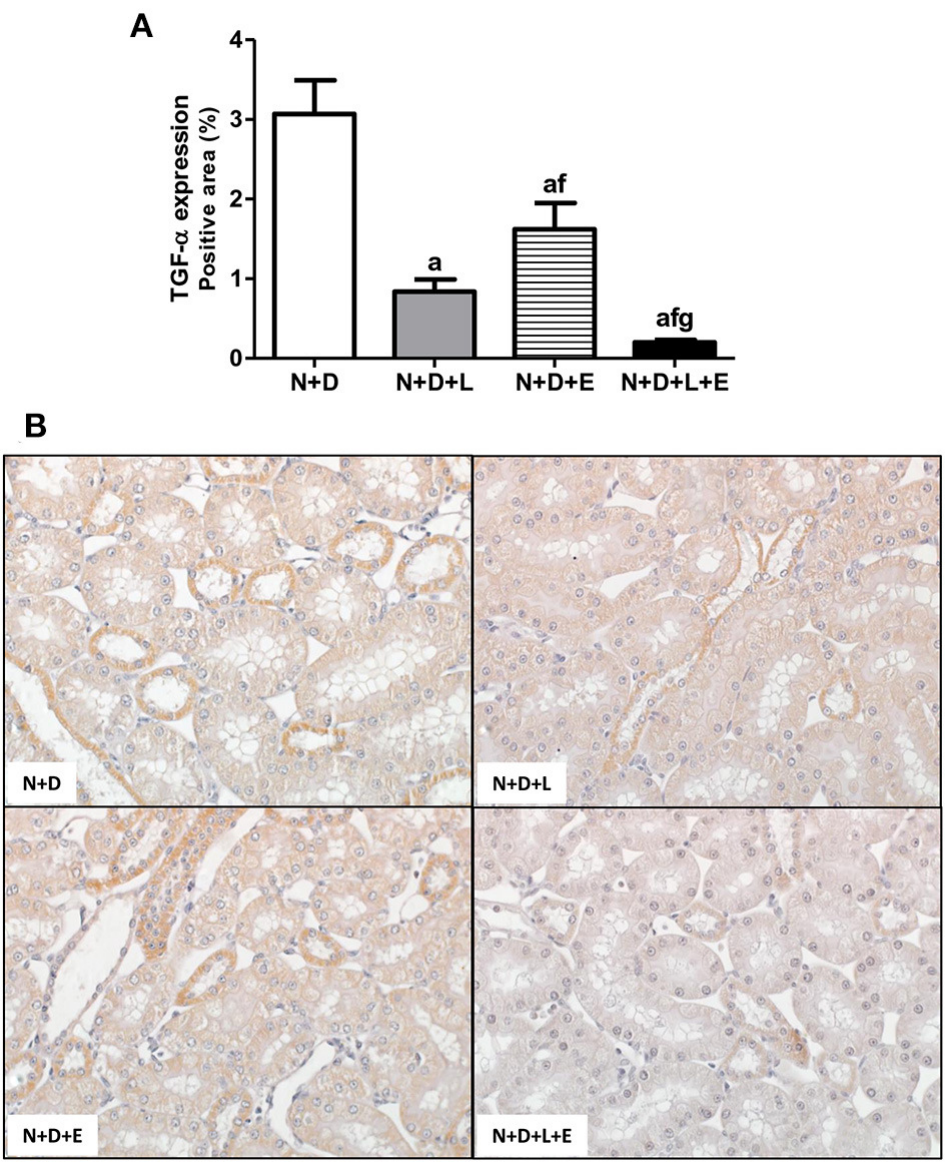
Immunohistochemical analysis of transforming growth factor α (TGF-α) expression in the renal cortex. **(A)** Bar graph of TGF-α expression evaluated 60 days after 5/6 nephrectomy in vitamin D-deficient rats treated with losartan, erlotinib or losartan+erlotinib. **(B)** Representative photomicrographs of immunostaining for TGF-α in the renal cortex from a N+D, N+D+L, N+D+E, and N+D+L+E rat (x400). Note that TGF-α expression is significantly lower in the N+D+L+E than in the other groups. Data are mean ± SEM. ^a^*p* < 0.001 vs. N+D; ^f^*p* < 0.05 vs. N+D+L; ^g^*p* < 0.001 vs. N+D+E. N, 5/6-nephrectomized rats; D, vitamin D-deficient rats; L, losartan-treated rats; E, erlotinib-treated rats; L+E, rats concomitantly treated with losartan+erlotinib. N+D (*n* = 10); N+D+L (*n* = 8); N+D+E (*n* = 7); N+D+L+E (*n* = 10).

**Figure 5:**
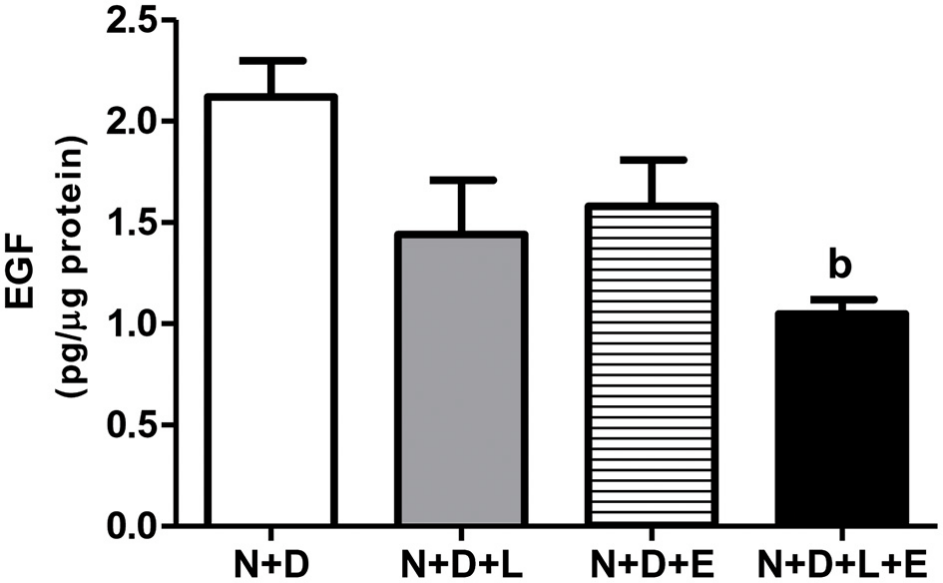
Quantitative amount of epidermal growth factor (EGF) in the renal cortex evaluated by ELISA 60 days after 5/6 nephrectomy in vitamin D-deficient rats treated with losartan, erlotinib or losartan+erlotinib. Data are mean ± SEM. ^b^*p* < 0.01 vs. N+D. N, 5/6-nephrectomized rats; D, vitamin D-deficient rats; L, losartan-treated rats; E, erlotinib-treated rats; L+E, rats concomitantly treated with losartan+erlotinib. N+D (*n* = 10); N+D+L (*n* = 8); N+D+E (*n* = 7); N+D+L+E (*n* = 10).

**Figure 6:**
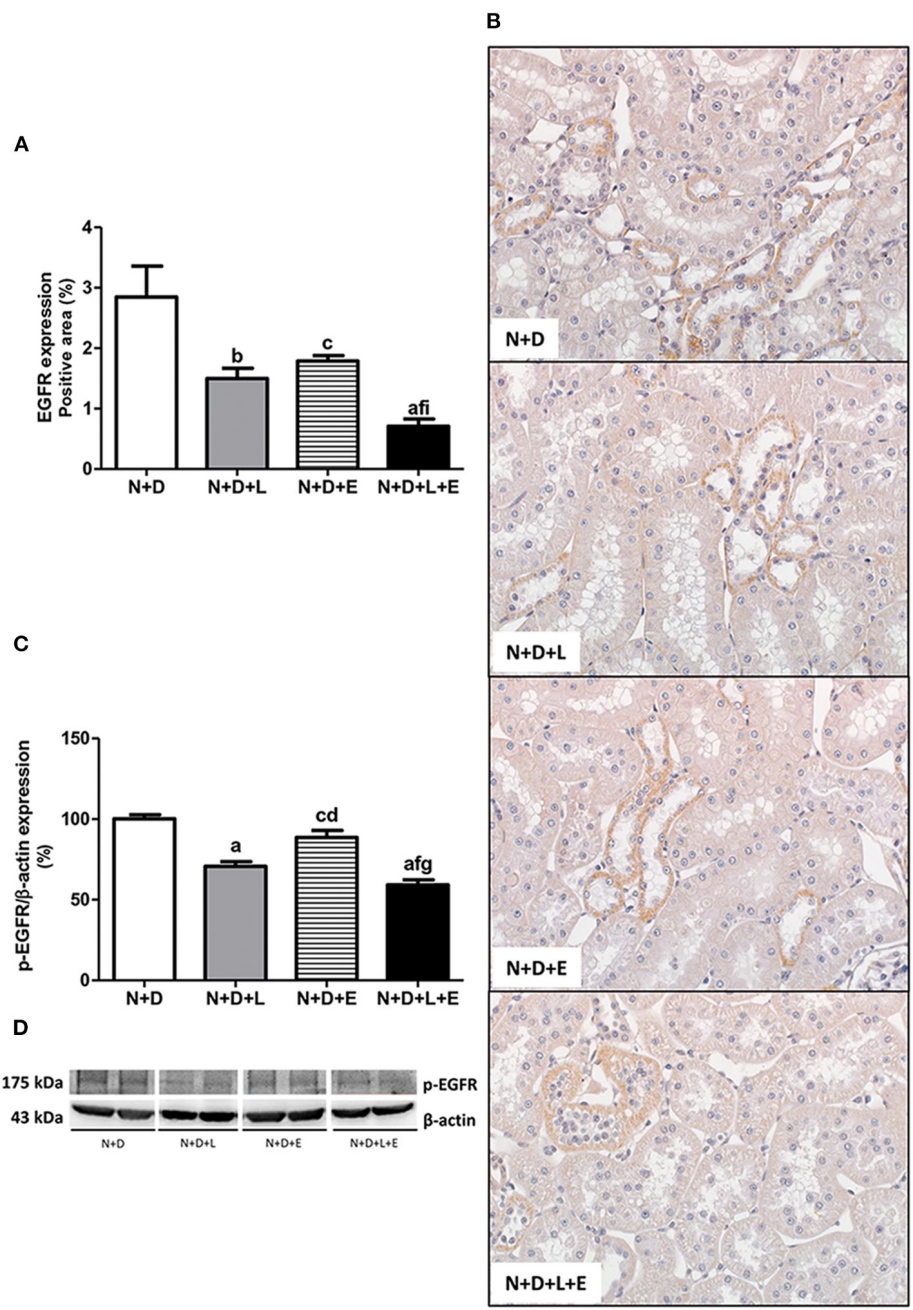
Assessment of both epidermal growth factor receptor (EGFR) and phospho-EGFR (p-EGFR) expression in the renal cortex evaluated 60 days after 5/6 nephrectomy in vitamin D-deficient rats treated with losartan, erlotinib or losartan+erlotinib. **(A)** Bar graph of immunohistochemical analysis of EGFR in the N+D, N+D+L, N+D+E, and N+D+L+E groups. **(B)** Representative photomicrographs of immunostaining for EGFR in the renal cortex from a N+D, N+D+L, N+D+E, and N+D+L+E rat (x400). Note that EGFR expression is significantly lower in the N+D+L+E than in the other groups. **(C)** Semiquantitative immunoblotting for p-EGFR with the respective densitometric analysis of samples. **(D)** Representative immunoblots which reacted with anti-p-EGFR revealing a 175 kDa band. Data are mean ± SEM. ^a^*p* < 0.001, ^b^*p* < 0.01, ^c^*p* < 0.05 vs. N+D; ^d^*p* < 0.001, ^f^*p* < 0.05 vs. N+D+L; ^g^*p* < 0.001, ^i^*p* < 0.05 vs. N+D+E. N, 5/6-nephrectomized rats; D, vitamin D-deficient rats; L, losartan-treated rats; E, erlotinib-treated rats; L+E, rats concomitantly treated with losartan+erlotinib. N+D (*n* = 10); N+D+L (*n* = 8); N+D+E (*n* = 7); N+D+L+E (*n* = 10).

After a simple evaluation of TGF-β amounts in the renal tissue, we attempted to study the effects of the monotherapies with losartan or erlotinib as well as the dual treatment losartan+erlotinib on the expression of the major elements of TACE-dependent EGFR activation pathway. Firstly, our evaluation of TACE (%) revealed a significant lower renal expression of this enzyme in the N+D+L and N+D+E groups than in the N+D group (Figure 3). Of note, the combined therapy losartan+erlotinib markedly prevented the expression of TACE in the N+D+L+E group compared to the other groups (Figure 3). Next, we demonstrated that the TGF-α renal expression (%) was significantly attenuated by monotherapies with losartan or erlotinib after comparing N+D+L and N+D+E groups with N+D group (Figure 4). Noteworthy, the dual treatment losartan+erlotinib provided an impressive prevention of TGF-α expression, almost abolishing the amounts of this cytokine in the renal epithelial cells of the distal tubular segments from N+D+L+E rats (Figure 4). Regarding EGF quantification, our results showed that only the double therapy losartan+erlotinib significantly lowered the renal expression of this factor in the N+D+L+E group compared to the other groups (Figure 5). Subsequently, our results from both IHC analysis (%) and Western Blotting (%) for EGFR and p-EGFR, respectively, showed that both monotherapies with losartan or erlotinib significantly lowered the expression of EGFR and p-EGFR in the N+D+L and N+D+E groups compared to the N+D group (Figure 6). Similar to TGF-α expression, the dual treatment losartan+erlotinib considerably prevented the expression of both EGFR and p-EGFR in the renal cortex of the N+D+L+E group compared to the other three groups (Figure 6). Thus, we noted that the combined therapy losartan+erlotinib was more effective in preventing the expression of EGFR and p-EGFR in the N+D+L+E group in comparison to the other groups (Figure 6). Of note, EGFR was prominently immunopositive in the renal epithelial cells of the distal tubular segments, including the loop of Henle, convoluted distal tubule and collecting ducts. Based on our results, it is possible to deduct that the combined therapy losartan+erlotinib was more effective in blocking the expression of the major elements of TACE-dependent EGFR activation pathway.

### The Association Losartan+Erlotinib Markedly Prevented the Expression of ECM Components and Markers of Phenotypic Change

**Figure 7:**
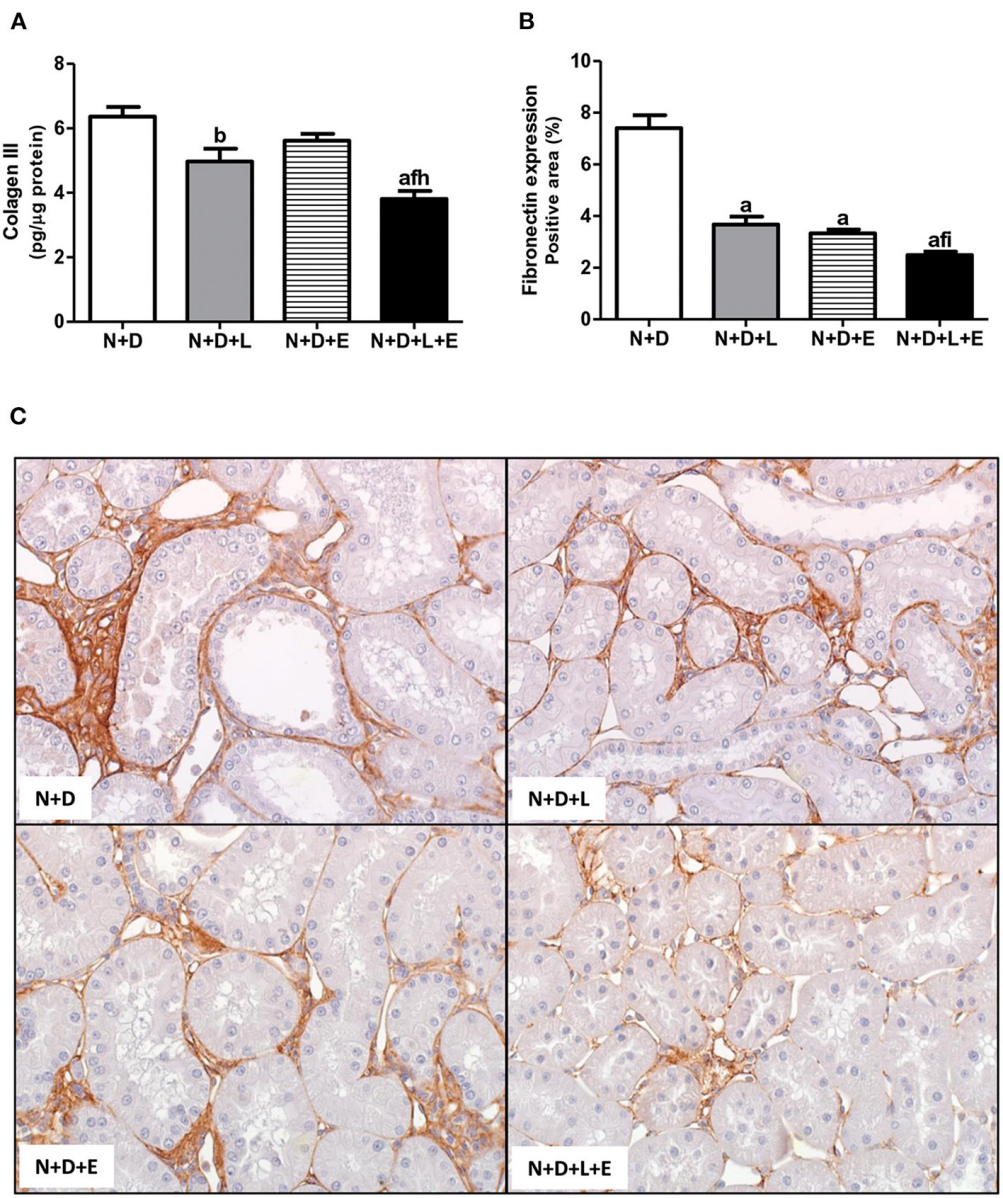
Analysis of extracellular matrix proteins expression in the renal cortex evaluated 60 days after 5/6 nephrectomy in vitamin D-deficient rats treated with losartan, erlotinib or losartan+erlotinib. **(A)** Quantitative amount of collagen III (Col III) evaluated by ELISA in N+D, N+D+L, N+D+E and N+D+L+E groups. **(B)** Bar graph of immunohistochemical analysis of fibronectin expression in N+D, N+D+L, N+D+E and N+D+L+E groups. **(C)** Representative photomicrographs of immunostaining for fibronectin in the renal cortex from a N+D, N+D+L, N+D+E and N+D+L+E rat (x400). Note that fibronectin expression is significantly lower in the N+D+L+E than in the other groups. ^a^*p* < 0.001, ^b^*p* < 0.01 vs. N+D; ^f^*p* < 0.05 vs. N+D+L; ^h^*p* < 0.01, ^i^*p* < 0.05 vs. N+D+E. N, 5/6-nephrectomized rats; D, vitamin D-deficient rats; L, losartan-treated rats; E, erlotinib-treated rats; L+E, rats concomitantly treated with losartan+erlotinib. N+D (*n* = 10); N+D+L (*n* = 8); N+D+E (*n* = 7); N+D+L+E (*n* = 10).

**Figure 8:**
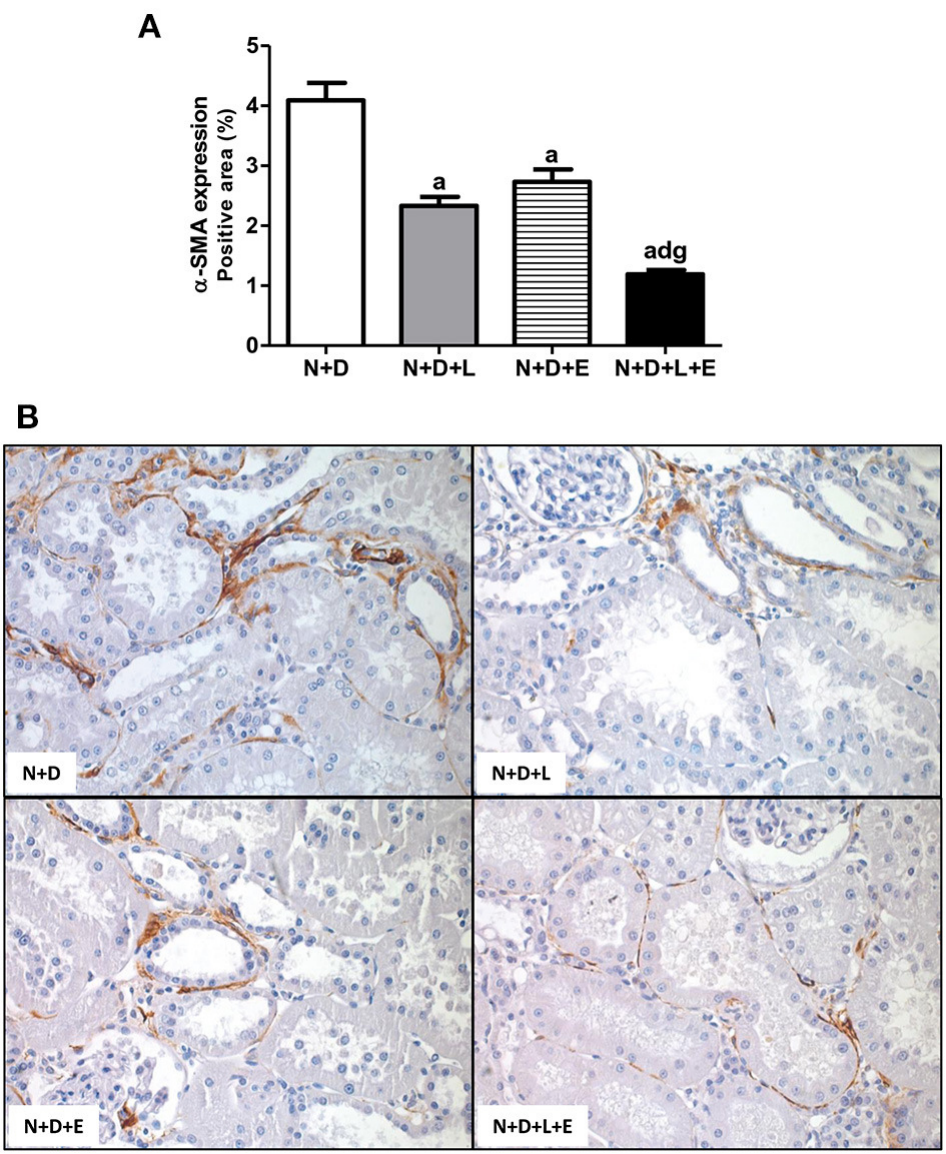
Immunohistochemical analysis of α-smooth muscle actin (α-SMA) expression in the renal cortex. **(A)** Bar graph of α-SMA expression evaluated 60 days after 5/6 nephrectomy in vitamin D-deficient rats treated with losartan, erlotinib or losartan+erlotinib. **(B)** Representative photomicrographs of immunostaining for α-SMA in the renal cortex from a N+D, N+D+L, N+D+E and N+D+L+E rat (x400). Note that α-SMA expression is significantly lower in the N+D+L+E than in the other groups. Data are mean ± SEM. ^a^*p* < 0.001 vs. N+D; ^d^*p* < 0.001 vs. N+D+L; ^g^*p* < 0.001 vs. N+D+E. N, 5/6-nephrectomized rats; D, vitamin D-deficient rats; L, losartan-treated rats; E, erlotinib-treated rats; L+E, rats concomitantly treated with losartan+erlotinib. N+D (*n* = 10); N+D+L (*n* = 8); N+D+E (*n* = 7); N+D+L+E (*n* = 10).

**Figure 9:**
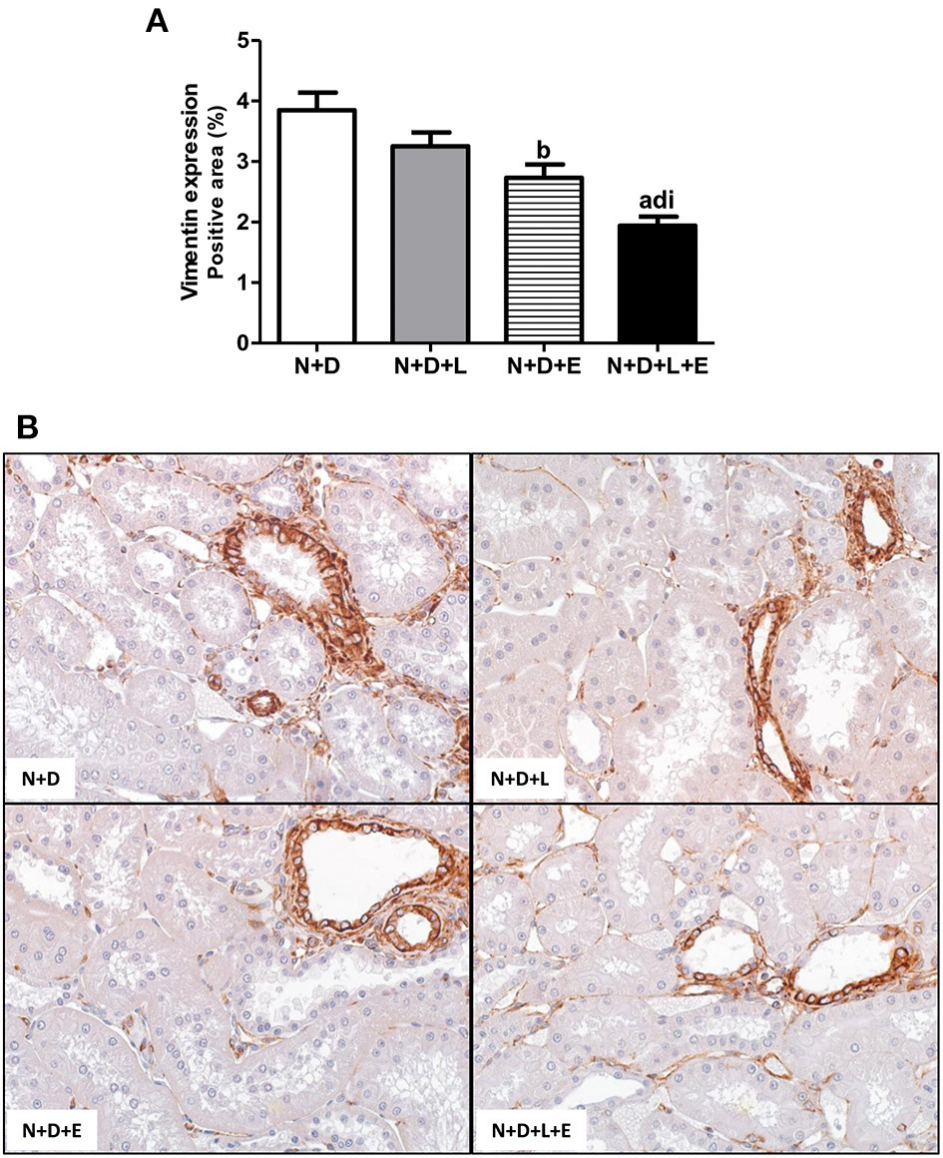
Immunohistochemical analysis of vimentin expression in the renal cortex. **(A)** Bar graph of vimentin expression evaluated 60 days after 5/6 nephrectomy in vitamin D- deficient rats treated with losartan, erlotinib or losartan+erlotinib. **(B)** Representative photomicrographs of immunostaining for vimentin in the renal cortex from a N+D, N+D+L, N+D+E, and N+D+L+E rat (x400). Note that vimentin expression is significantly lower in the N+D+L+E than in the other groups. Data are mean ± SEM. ^a^*p* < 0.001, ^b^*p* < 0.01 vs. N+D; ^d^*p* < 0.001 vs. N+D+L; ^i^*p* < 0.05 vs. N+D+E. N, 5/6-nephrectomized rats; D, vitamin D-deficient rats; L, losartan-treated rats; E, erlotinib-treated rats; L+E, rats concomitantly treated with losartan+erlotinib. N+D (*n* = 10); N+D+L (*n* = 8); N+D+E (*n* = 7); N+D+L+E (*n* = 10).

As previously described, our data on morphological changes and pathways of RFF (TGF-β and TACE-dependent EGFR activation) demonstrated that the double treatment losartan+erlotinib was more efficient in blocking those pathways. Moreover, the blockade of those pathways was directly associated with a significant decrease on interstitial enlargement. The expansion of tubulointerstitial compartment and RFF are complex processes and involve the production and secretion of various ECM components. Thus, our next step was to investigate the expression of two ECM proteins, including fibronectin and Col III in the renal tissue. Our results related to fibronectin renal expression (%) showed that the monotherapies with losartan or erlotinib significantly decreased the expression of this ECM protein in the N+D+L and N+D+E groups compared to the N+D group (Figure 7). Differently from fibronectin, only treatment with losartan alone significantly lowered the renal expression of Col III (pg/μg prot) in the N+D+L group compared to the N+D group (Figure 7). Most importantly, the combined treatment losartan+erlotinib markedly prevented the renal expression of both fibronectin and Col III in the N+D+L+E group compared to the other groups (Figure 7). Regarding the presence of phenotypic alteration of renal tubular cells, we found a significant lower renal expression of α-SMA (%) in the N+D+L and N+D+E groups compared to the N+D group (Figure 8). In addition, the monotherapy with erlotinib significantly reduced the renal expression of vimentin (%) in the N+D+E group compared to the N+D group (Figure 9). Most significantly, the combined treatment losartan+erlotinib prevented the renal expression of both α-SMA and vimentin in the N+D+L+E rats compared to those from other groups (Figures 8, 9). Therefore, the dual treatment losartan+erlotinib was more efficient in the prevention of ECM production and phenotypic alteration after EMT process.

### Effects of Losartan+Erlotinib Treatment on Tubulointerstitial Inflammatory Cell Infiltrate and Expression of M2 Macrophages

**Figure 10:**
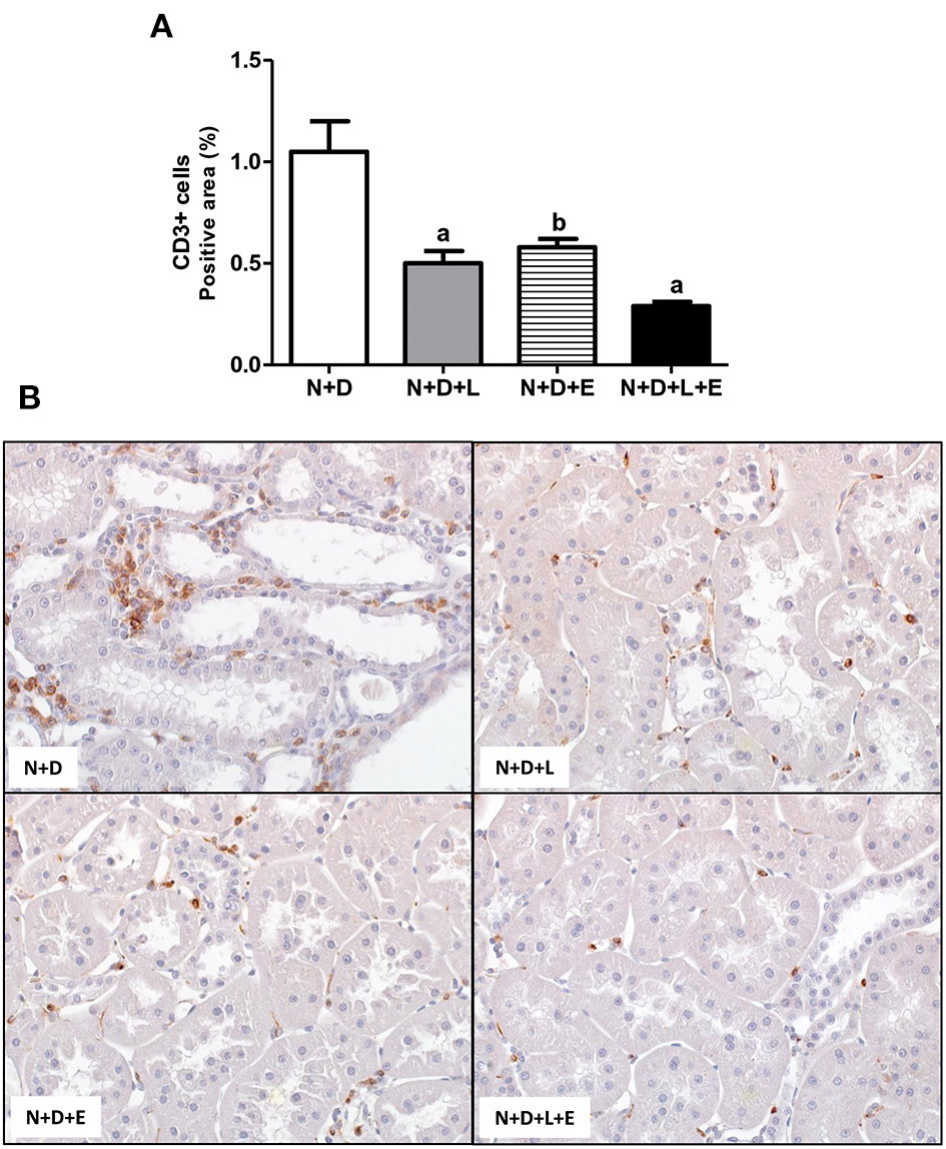
Immunohistochemical analysis of CD3+ cells (T cells) in the renal cortex. **(A)** Bar graph of CD3+ cells expression evaluated 60 days after 5/6 nephrectomy in vitamin D-deficient rats treated with losartan, erlotinib or losartan+erlotinib. **(B)** Representative photomicrographs of immunostaining for CD3+ cells in the renal cortex from a N+D, N+D+L, N+D+E, and N+D+L+E rat (x400). Data are mean ± SEM. ^a^*p* < 0.001, ^b^*p* < 0.01 vs. N+D. N, 5/6-nephrectomized rats; D, vitamin D-deficient rats; L, losartan-treated rats; E, erlotinib-treated rats; L+E, rats concomitantly treated with losartan+erlotinib. N+D (*n* = 10); N+D+L (*n* = 8); N+D+E (*n* = 7); N+D+L+E (*n* = 10).

**Figure 11:**
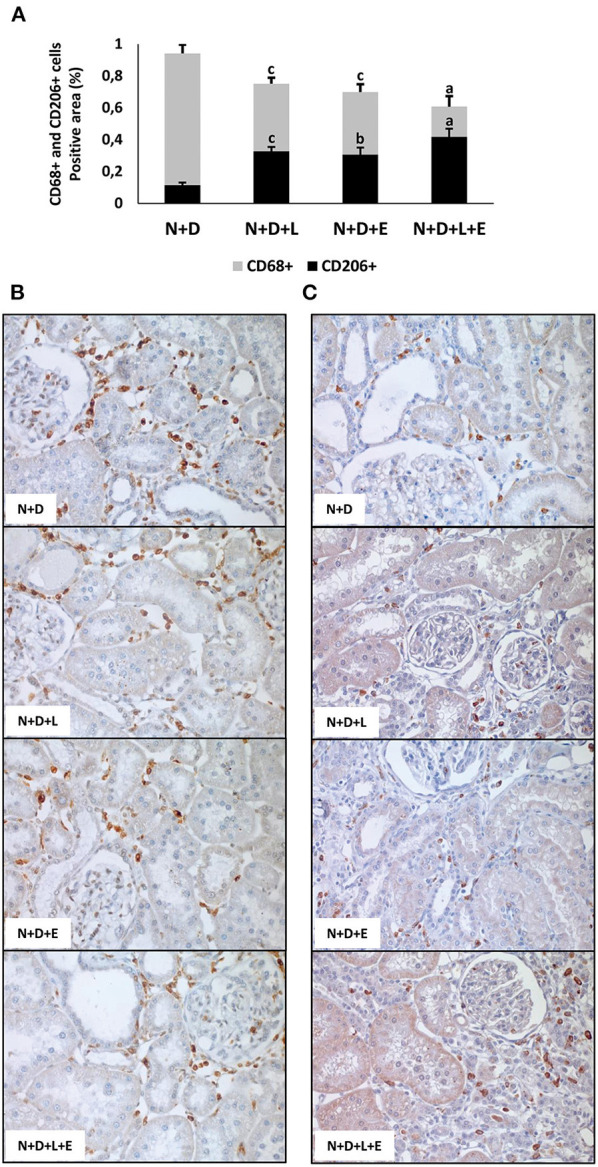
Immunohistochemical analysis of CD68+ cells (M1+M2 macrophages) and CD206+ cells (M2 macrophages) expression in the renal cortex evaluated 60 days after 5/6 nephrectomy in vitamin D-deficient rats treated with losartan, erlotinib or losartan+erlotinib. **(A)** Bar graph regarding the proportion of CD206+ cells in relation to the amount of CD68+ cells in N+D, N+D+L, N+D+E, and N+D+L+E groups. **(B)** Representative photomicrographs of immunostaining for CD68+ cells in the renal cortex from a N+D, N+D+L, N+D+E, and N+D+L+E rat (x400). **(C)** Representative photomicrographs of immunostaining for CD206+ cells in the renal cortex from a N+D, N+D+L, N+D+E, and N+D+L+E rat (x400). Note the higher expression of M2 macrophage subtype in the N+D+L, N+D+E, and N+D+L+E in comparison to the N+D. Data are mean ± SEM. ^a^*p* < 0.001, ^b^*p* < 0.01, ^c^*p* < 0.05 vs. N+D. N, 5/6-nephrectomized rats; D, vitamin D-deficient rats; L, losartan-treated rats; E, erlotinib-treated rats; L+E, rats concomitantly treated with losartan+erlotinib. N+D (*n* = 10); N+D+L (*n* = 8); N+D+E (*n* = 7); N+D+L+E (*n* = 10).

As mentioned before, one of the major features of the enlargement of tubulointerstitial compartment in 5/6 nephrectomy model is the presence of inflammatory cell infiltrate in the renal cortex. Initially, we assessed the renal expression of CD68+cells (macrophages) and CD3+(T cells) in order to estimate the amounts of these inflammatory cells in the renal cortex. As shown in Figures 10, 11, the monotherapies with losartan or erlotinib as well as the combined treatment losartan+erlotinib significantly reduced the renal expression (%) of both CD68+ and CD3+ cells in N+D+L, N+D+E and N+D+L+E groups compared to N+D group. Based on the different characteristics of macrophages, our next step was to investigate the population of CD206+ cells (M2 macrophages), which are generally related to tissue repair (29, 30). As demonstrated in Figure 11, the percentage of CD206+ cells (%) was significantly higher in the rats under losartan, erlotinib or losartan+erlotinib treatments than in the N+D rats. Thus, in an overall analysis of our data, we could infer that both monotherapies with losartan or erlotinib as well as the combined treatment losartan+erlotinib not only contributed to reduce the renal expression of inflammatory cells (CD68+ and CD3+) but also improved the renal expression of M2 macrophages (CD206+) usually related to tissue repair.

## Discussion

Renal fibrosis formation is the major feature underlying the progression of CKD to ESRD. Several studies have been showing an increasing incidence and prevalence of CKD, which is usually aggravated by risk factors such as cardiovascular/hemodynamic diseases, infections and diabetes (1, 2, 4). In addition to the well-known risk factors related to the CKD progression, other studies have been highlighting the hypovitaminosis D as a non-traditional aggravating factor for renal diseases (4–8, 31, 32). Previously, we demonstrated that VDD not only impaired the recovery but also accelerated the renal disease progression, exerting an important role on the process of RFF in different experimental models (4–9, 31). Although most studies have been focusing their efforts on the mechanisms underlying RFF, there is a lack of effective anti-fibrotic therapies which could efficiently block or reverse the progression of CKD. In this present study, we demonstrated that the association between losartan, a well-known AT1R antagonist, with erlotinib, a blocker of EGFR activity, successfully provided functional and histomorphological benefits in a CKD model (5/6 nephrectomy) aggravated by VDD. In addition to an attenuation of GFR and MAP, we observed a lower inflammatory cell infiltrate and a prevention regarding the expression of elements related to RFF, such as TGF-β1, TACE, TGF-α, EGF, and EGFR in the N+D+L+E group. Most importantly, N+D+L+E group also presented a lower expression of ECM components and markers of phenotypic alteration associated with a significant decrease concerning the expansion of FIA of the tubulointerstitial compartment.

Clinical studies have been demonstrating that hypovitaminosis D is common in patients with CKD even in the early stages, which usually increases with the progression of CKD (4, 33, 34). Moreover, there is a common understanding that low levels of 25(OH)D is related to a negative calcium balance and secondary hyperparathyroidism. Thus, in addition to 25(OH)D, we also evaluated the plasma levels of intact PTH, FGF-23, calcium and phosphorus. As described earlier, plasma levels of phosphorus and calcium did not change among the experimental groups. Associated to the undetectable 25(OH)D levels, we found significant lower PTH levels in the N+D+L+E group than in the N+D group and only a downward trend in PTH levels in the other groups. It has been experimentally demonstrated that the inhibition of EGFR by erlotinib attenuated parathyroid hyperplasia and the loss of VDR, restoring the response to PTH (35). There are multiple loops of negative feedback among the main regulators of bone mineral metabolism, including PTH, FGF-23 and vitamin D (36–39). In our experimental protocol, there were some variables that may have interfered in that dynamics. It is important to note that our experimental model reflected a CKD at a moderate stage, therefore, we did not expect to find significant changes in bone mineral metabolism. In addition, the vitamin D-free diet offered to our rats also contained low levels of calcium (0.4%) and phosphate (0.4%). Based on a previous study, we observed a reduction of ~8% on the plasma calcium levels in rats under VDD also euthanized 60 days after 5/6 nephrectomy (4). Low levels of vitamin D associated with hypocalcemia are known to reduce FGF-23 levels and can directly and indirectly stimulate PTH production (36–39). Here, our treated groups may have shown an improvement in PTH due to an amelioration in GFR. However, we cannot exclude the possibility that the double therapy losartan+erlotinib may have exerted an influence on PTH levels. Therefore, further studies are needed for a better understanding along the changes in bone mineral metabolism attributed to the synergistic effect of losartan+erlotinib treatment.

Subtotally nephrectomized rats usually present an impaired renal function, systemic hypertension and proteinuria (4, 40, 41). These functional changes are usually accompanied by increased FIA, as well as increased inflammatory infiltrate (4, 40). Studies have been showing that those renal structural changes characteristic of 5/6 nephrectomy are a consequence of mechanical stress, caused by glomerular hypertension and hypertrophy (42, 43), infiltration/proliferation of inflammatory cells, and accumulation of ECM (43, 44). In addition, some reports state that the distension of the glomerular walls promoted by intracapillary hypertension may trigger the local release of cytokines, growth factors and, particularly, AII (45, 46). These findings are supported by experimental and clinical observations over 30 years, which show that progressive nephropathies can be attenuated, but not completely blocked, by early treatment with AT1R antagonists or angiotensin-converting enzyme inhibitors (40, 47–49). Previous studies have been demonstrating that VDD can upregulate the RAAS activity, reinforcing the role of vitamin D status on the renal function preservation and blood pressure control (4, 50–52). Firstly, our functional data showed that N+D+L and N+D+L+E, both losartan-treated groups, presented a recovery of GFR compared to N+D group. Thus, our findings support the important role of losartan as a renoprotective drug and suggest a hemodynamic and functional benefits of this medication when used in the earlier stages of CKD. In regard to erlotinib alone, we observed only a tendency in N+D+E group to recover the renal function, without significance. Conversely, Yamamoto et al. (20) reported an improvement in renal function, based on serum creatinine levels, in 5/6-nephrectomized rats also treated with erlotinib. However, different from our protocol, the authors used a higher dose of erlotinib (20 mg/kg) in the absence of hypovitaminosis D. Concerning our blood pressure results, we found remarkable and expected lower levels of MAP in both losartan-treated groups and a significant decreasing in this parameter in N+D+E group as well. In addition, we observed a significant lower plasma aldosterone levels in N+D+L and N+D+L+E groups. In accordance to the aforementioned studies, the joint analysis of our data shows the importance of AII antagonists usage on blood pressure control (47, 53, 54) and reinforces the role of RAAS in the increase of MAP under hypovitaminosis D, which have already been described by our research group (4–6). However, further studies are necessary in order to elucidate the participation of erlotinib alone on blood pressure control.

Chronic kidney disease is usually characterized by a progressive reduction in renal function and structural kidney damage (4, 55). Regardless of cause, tubulointerstitial fibrosis is the main process which progressively affects kidneys in the course of CKD (16, 56). In the present study, we predictably observed renal morphological changes such as necrosis, tubular atrophy and dilatation, inflammatory cell infiltrate and fibrosis in the kidneys, which are well-described features from 5/6 nephrectomy model. Associated with those alterations, we found a remarkable enlargement of the tubulointerstitial compartment (increased FIA), predominantly in the renal cortex of the N+D group. Most importantly, N+D+L and N+D+E groups and mainly N+D+L+E group, presented an impressive reduction concerning FIA. Corroborating our FIA findings, both monotherapies with losartan or erlotinib, and particularly the dual treatment losartan+erlotinib, significantly reduced proteinuria. Taken together, probably the improvement found in the morphological changes in both erlotinib-treated groups was not only associated with hemodynamic factors, but also with a modulation of RFF signaling pathways.

Currently no effective therapy is available to completely arrest RFF, which could contribute to slow the progression of CKD. A number of important studies have been clarifying the cellular and molecular mechanisms related to RFF, especially those associated with the TGF-β canonical signaling *via Smads* (18, 56). As described earlier, TGF-β has been implicated to be a major regulatory profibrotic cytokine in CKD. In addition, many injurious stimuli converge on the TGF-β pathway, which has context-dependent pleiotropic effects and interacts with several related RFF pathways (18). Many reports have been suggesting that EGFR is associated with TGF-β-dependent RFF and EMT as well (20, 24, 25, 57). EGFR is expressed in various cell types and the multiple signaling pathways of this receptor highlights its importance regarding cell survival, proliferation and migration (19, 20). Moreover, some experimental studies have been supporting a role for EGFR in RFF, demonstrating for instance, that the overexpression of a dominant negative isoform of EGFR protected mice from tubulointerstitial damage in 5/6 nephrectomy, chronic AII infusion and ischemia/reperfusion injury (20, 22, 58, 59).

Several lines of evidence have demonstrated the role of RAAS, particularly AII, on the morphological changes of the renal structure (22, 26, 60, 61). The mechanisms by which AII promotes RFF remain not completely clear (26). It is known that AII stimulates both TGF-β signaling pathway and activates EGFR, however, the relative contribution of each pathway regarding fibrogenesis is not fully understood (24, 60, 61). Based on that, we attempted to use a double therapy including losartan+erlotinib in order to block TACE-dependent EGFR activation, one of the EGFR pathways related to RFF. Briefly, losartan would act binding to AT1R, blocking the activation of TACE and subsequent releasing of TGF-α, which would bind to EGFR (21). On the other hand, erlotinib acting as a TKI, would inhibit the EGFR autophosphorylation, blocking the downstream signaling transduction related to cell growth, proliferation and migration (20, 62). As previously described, our results showed that the dual treatment losartan+erlotinib efficiently prevented the expression of TGF-α and its sheddase TACE, and both EGFR and p-EGFR expressions as well, which were associated with a remarkable decrease of EGF plasma levels in the N+D+L+E group. Therefore, our data show that, in addition to the efficient blockade observed in TGF-β expression, losartan+erlotinib treatment also prevented the activation of the EGFR pathway, resulting in a lower rate of RFF observed in the N+D+L+E group. In 2012, Chen et al. (24) showed a crucial role of tubular epithelial cells (TEC) on EGFR activation in TGF-β-dependent tubulointerstitial fibrosis. The authors identified that TGF-β-mediated tissue fibrosis depends on a persistent feed-forward mechanism of EGFR/ERK activation through an unexpected signaling pathway. This finding highlighted EGFR as a potential therapeutic target involved in the modulation of fibrogenesis (24). Similar to our study, Lautrette et al. (22) evaluated the link between EGFR and AII showing that the specific deletion of TGF-α or the inhibition of TGF-α cleavage by using a specific TACE inhibitor (WTACE2) and losartan significantly reduced renal damage. The authors suggested a role of TGF-α as a key factor between AII signaling and EGFR transactivation during AII-induced nephropathy, regardless of blood pressure levels (22). Recently, Yamamoto et al. (20) showed that EGFR blocking by erlotinib alone protected against RFF in 5/6-nephrectomized rats *via* inhibition of Akt and ERK 1/2 signaling pathways, which were closely associated with renal fibrosis. In our study, we also observed an important effect of erlotinib alone on the modulation of interstitial enlargement, with a reduction of ~35% in the FIA from N+D+E group. However, losartan+erlotinib treatment provided a reduction of ~53% in FIA evaluation in the N+D+L+E group compared to the N+D. Thus, our data suggest that the dual therapy losartan+erlotinib could represent a prospective strategy to slow RFF in CKD by blocking both TGF-β and EGFR pathways.

The pathogenesis of renal fibrosis is characterized by proliferation of activated fibroblasts (myofibroblasts) and overproduction of ECM proteins such as collagens type I, III, V, VII, and XV, as well as the adhesive glycoprotein fibronectin (25, 56, 63). Upon activation, several cell types including macrophages, T cells and TEC produce profibrotic mediators such as TGF-β, CTGF, PDGF, and EGF, which stimulate mesenchymal cells to turn into myofibroblasts after EMT process (18). In our study, associated with a successful blockade of TGF-β, TGF-α, and EGF expressions, we observed a prevention of fibronectin and Col III amounts in the renal tissue from N+D+L+E rats. In addition, our data regarding markers of phenotypic alteration demonstrated a remarkable decrease in the renal expression of α-SMA and vimentin in the N+D+L+E rats. It has been shown a critical relationship between the activation of damaged TEC and the role of EGFR signaling in TGF-β-dependent RFF (20, 24). In addition to TGF-β1, other profibrotic factors, including AII and aldosterone, can induce EGFR transactivation which subsequently activate downstream pathways related to cell proliferation and ECM production (23, 25). Since the double treatment losartan+erlotinib was able to successfully prevent both ECM production and phenotypic alteration, our data suggest that EGFR could be considered as a key element involved in the effects of various profibrotic factors associated to our model of CKD. It has been described that, in contrast to a transient activation/expression of EGFR in AKI, chronic injury to the kidneys can induce a persistent EGFR expression and phosphorylation (25). Furthermore, the mechanisms underlying this possible sustained EGFR activation could be not only associated with an increased production of EGFR ligands (i.e., TGF-α) (25, 58), but also with some non-EGFR ligands, such as AII and endothelin (22, 25, 64).

Inflammation exerts a pivotal role in initiation and maintenance of kidney injury and a lower inflammatory response is usually associated with a mitigation of RFF. Inflammatory cells such as macrophages and T cells play an important role in tissue homeostasis and immune responses, especially in the course of renal diseases (4). The participation of the EGFR pathway and the effects of erlotinib in inflammation have been stated (20, 65). In this present study, we observed an increased expression of both CD3+ and CD68+ cells in the renal cortex of N+D group as an indicative of a more severe inflammation. On the other hand, the double therapy losartan+erlotinib resulted in not only a slight improvement regarding the lower inflammatory cell infiltrate but also in a higher expression of CD206+ cells in comparison to losartan or erlotinib monotherapies. This is an important result concerning both anti-inflammatory and tissue repair functions related to M2 macrophages, especially in active renal lesions which are persistent in 5/6 nephrectomy model (4). Previous studies which targeted genetic or pharmacological blockade of both EGFR and its ligands reported a modulation of renal inflammation, with a diminished number of inflammatory cells (19, 65–67). Recently, Yamamoto et al. (20) also demonstrated that treatment with erlotinib alone attenuated the tubulointerstitial infiltration of macrophages in 5/6-nephrectomized rats. However, the mechanisms by which EGFR regulates the production of chemoattractant factors for inflammatory cells remain not fully understood. A possible explanation could be that EGFR, like TGF-β, regulates the production of cytokines through cell cycle arrest in G2/M phase (25). Moreover, EGFR may be linked to the production of these cytokines by acting on macrophages and other inflammatory cells, since several inflammatory cells are accumulated in the kidney after injury (25).

In conclusion, 5/6 nephrectomy is a model of CKD which progresses to irreversible alterations on function and structure of the kidney. Under hypovitaminosis D, these alterations are aggravated due to an acceleration observed on the renal disease progression. Our data demonstrated that the double treatment losartan+erlotinib not only blocked the TACE-dependent EGF receptor activation but also prevented the expression of TGF-β, protecting against RFF in 5/6-nephrectomized rats under VDD. This renoprotection by losartan+erlotinib was corroborated by a lower expression of ECM proteins and markers of phenotypic alteration as well as by a lesser inflammatory cell infiltrate observed in the kidneys from N+D+L+E rats. Although erlotinib alone has been emerging as a renoprotective drug, its association with losartan should be considered as a potential therapeutic strategy on the modulation of RFF.

## Data Availability Statement

The original contributions generated in the study are included in the article/supplementary materials, further inquiries can be directed to the corresponding author.

## Ethics Statement

The animal study was reviewed and approved by Research Ethics Committee (CEUA, registration 122/16) of the University of São Paulo.

## Author Contributions

JG, DC, AB, AS, MS, and RV conceived and designed the experiments. JG, DC, AB, MS, and RV performed the experiments. JG, DC, AB, and RV analyzed the data and contributed to the writing of the manuscript. All authors reviewed the manuscript.

## Conflict of Interest

The authors declare that the research was conducted in the absence of any commercial or financial relationships that could be construed as a potential conflict of interest.
